# Novel Pectate Lyase Genes of *Heterodera glycines* Play Key Roles in the Early Stage of Parasitism

**DOI:** 10.1371/journal.pone.0149959

**Published:** 2016-03-01

**Authors:** Huan Peng, Jiangkuan Cui, Haibo Long, Wenkun Huang, Lingan Kong, Shiming Liu, Wenting He, Xianqi Hu, Deliang Peng

**Affiliations:** 1 State Key Laboratory for Biology of Plant Diseases and Insect Pests, Institute of Plant Protection, Chinese Academy of Agricultural Sciences, Beijing, China; 2 Key Laboratory of Pests Comprehensive Governance for Tropical crops, Ministry of Agriculture, P. R. China, Environment and Plant Protection Institute, Chinese Academy of Tropical Agricultural Science, Danzhou, China; 3 The National Engineering Research Center of Agri-biodiversity Applied Technologies, Yunnan Agricultural University, Kunming, China; James Hutton Institute, UNITED KINGDOM

## Abstract

Pectate lyases are known to play a key role in pectin degradation by catalyzing the random cleavage of internal polymer linkages (endo-pectinases). In this paper, four novel cDNAs, designated *Hg-pel-3*, *Hg-pel-4*, *Hg-pel-6* and *Hg-pel-7*, that encode pectate lyases were cloned and characterized from the soybean cyst nematode, *Heterodera glycines*. The predicted protein sequences of HG-PEL-3, HG-PEL-4 and HG-PEL-6 differed significantly in both their amino acid sequences and their genomic structures from other pectate lyases of *H*. *glycines* (HG-PEL-1, HG-PEL-2 and HG-PEL-7). A phylogenetic study revealed that the pectate lyase proteins of *H*. *glycines* are clustered into distinct clades and have distinct numbers and positioning of introns, which suggests that the pectate lyase genes of *H*. *glycines* may have evolved from at least two ancestral genes. A Southern blot analysis revealed that multiple *Hg-pel-6*-like genes were present in the *H*. *glycines* genome. *In situ* hybridization showed that four novel pectate lyases (*Hg-pel-3*, *Hg-pel-4*, *Hg-pel-6* and *Hg-pel-7)* were actively transcribed in the subventral esophageal gland cells. A semi-quantitative RT-PCR assay supported the finding that the expression of these genes was strong in the egg, pre-parasitic second-stage juvenile (J2) and early parasitic J2 stages and that it declined in further developmental stages of the nematode. This expression pattern suggests that these proteins play a role in the migratory phase of the nematode life cycle. Knocking down *Hg-pel-6* using *in vitro* RNA interference resulted in a 46.9% reduction of the number of nematodes that invaded the plants and a 61.5% suppression of the development of *H*. *glycines* females within roots compared to the GFP-dsRNA control. Plant host-derived RNAi induced the silencing of the *Hg-pel-6*gene, which significantly reduced the nematode infection levels at 7 Days post inoculation (dpi). Similarly, this procedure reduced the number of female adults at 40 dpi, which suggests the important roles of this gene in the early stages of parasitism. Our combined data suggest that two types of pectate lyases are present in the *H*. *glycines* genome and may have different roles during infection.

## Introduction

*Heterodera glycines* Ichinohe, the soybean cyst nematode, is an obligatory endo-parasitic pathogen that is consistently the most damaging pest of soybean plants [1,2]. Recent estimates of the annual production losses caused by *H*. *glycines* range from $460 to $818 million in the US alone [3]. In China, the annual economic losses associated with *H*. *glycines* have been estimated to be $120 million [4]. Infective second-stage juveniles (J2) of *H*. *glycines* penetrate the soybean root tips and migrate intracellularly to the vascular cylinder to establish a permanent feeding site (syncytium) for its subsequent sedentary stages [5]. For plant parasitic nematodes (PPNs), the cell wall, which is primarily composed of pectin and cellulose, represents a formidable barrier to penetration and migration [6–7]. The synergistic effect of several enzymes is necessary for the degradation of pectin. These enzymes can be divided into the following two main groups: pectin esterases, which remove the methoxyl groups from pectin, and depolymerases (hydrolases and lyases), which cleave the back bones among galacturonate units [8]. For pectin degradation, two types of depolymerase, which include pectate lyase and polygalacturonase, have been isolated and characterized in PPNs. However, the pectin esterases that act on methylated pectin have not yet been recorded from PPNs [6].

The first pectate lyases from animal were discovered in the potato cyst nematode, *Globodera rostochiensis* [8]. Since then, pectate lyases have been isolated from several sedentary PPNs, such as species of *Heterodera*, *Globodera*, and *Meloidogyne* [9–13], and from migratory phytoparasitic nematodes, such as *Bursaphelenchus xylophilus* [14] and *Aphelenchus avenae* [15]. Subsequently, 15, 30 and 22 putative pectate lyases were predicted from the genome sequences of *B*. *xylophilus* [16], *M*. *incognita* [17] and *M*. *hapla* [18], respectively. These proteins are secreted by a pair of gland cells and are released into the plant tissue through the stylet of the nematode [19]. Pectate lyases, combined with a cocktail of cell wall-modifying enzymes (e.g., cellulases and hemicellulases), are thought to soften and degrade the structure of plant cell walls during nematode migration [6]. One of the two pectate lyases present in *H*. *schachtii* was silenced by RNAi, which resulted in fewer infections [13]. Moreover, the transient expression of *Gr-pel2* in *Nicotiana benthamiana* leaves resulted in severe malformations of the infiltrated tissues at six dpi [12], indicating that pectate lyases play an essential role in nematode-plant interactions. However, an RNAi of *hg-pel-2* in *H*. *glycines* caused a change in the sexual fate of the nematodes, which favored the male development but did not suppress the number of parasites in the root [20].

Here, four novel genes that encode pectate lyases were isolated from the soybean cyst nematode *H*. *glycines*. Furthermore, we analyzed the gene structure, expression characterizations and functional role of these novel pectate lyases.

## Results

### Identification of pectate lyases

Four ESTs similar to the pectate lyase of *H*. *schachtii* were identified in an EST dataset of *H*. *glycines*. Based on these sequences, four full-length cDNAs were obtained by the rapid amplification of cDNA ends (RACE). The sequence information of the four novel genes is shown in Table 1.

**Table 1:** Sequence information of the four novel pectate lyases.

Gene name	GenBank accession	Full length cDNA excluding the poly (dA) tail (bp)	Putative ORF (bp)	Predicted protein (amino acids)	Molecular weight(kDa)
*Hg-pel-3*	HQ123255	926	765	254	29.99
*Hg-pel-4*	HQ123256	934	768	255	29.72
*Hg-pel-6*	HQ123258	929	762	253	29.86
*Hg-pel-7*	HQ123259	1,007	792	263	27.96

**Fig 1:**
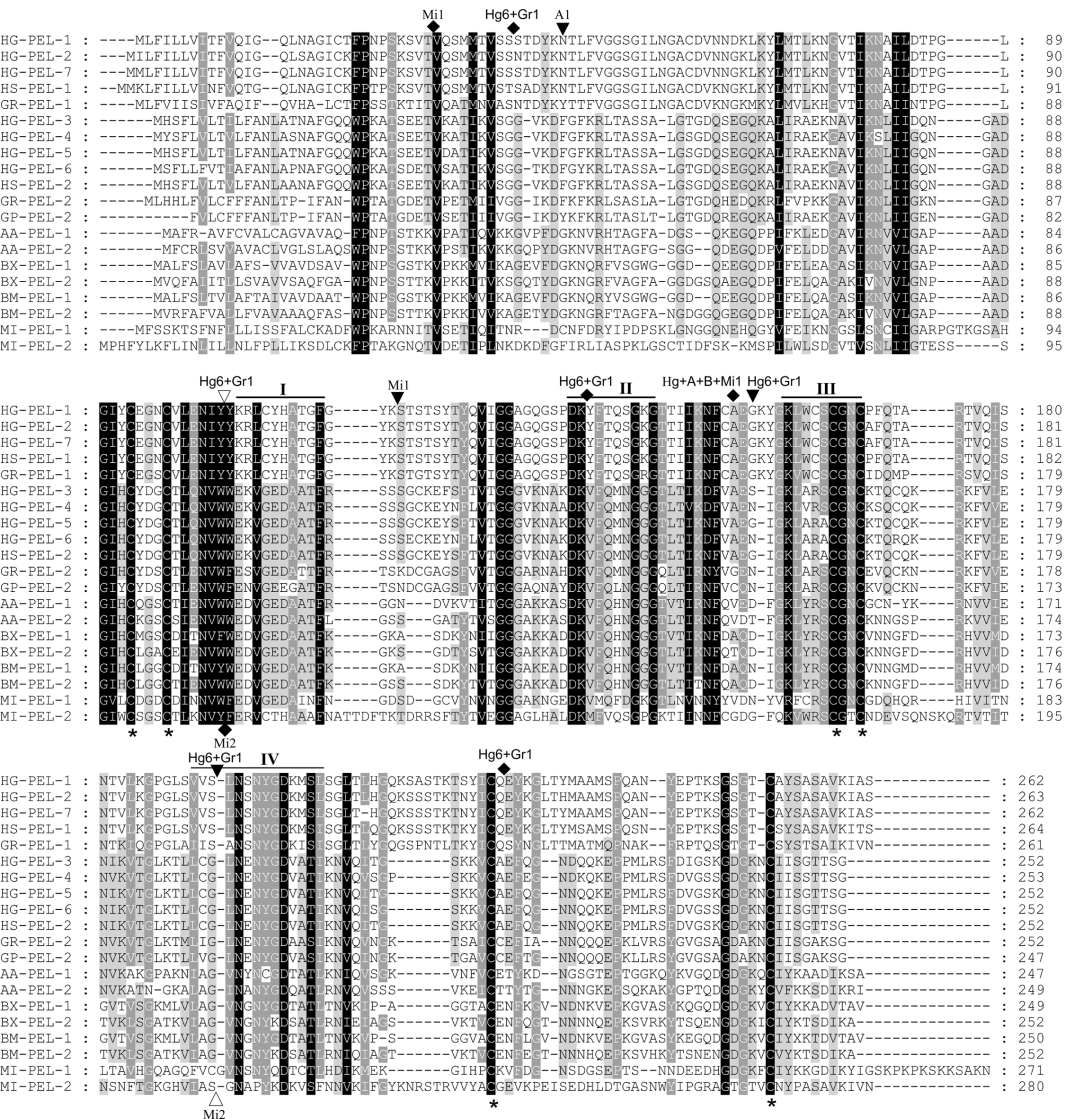
Alignment of the putative protein sequences of the pectate lyases that belong to the polysaccharide lyase family 3. HG-PEL-1 [AAK08974], HG-PEL-2 [AAM74954] and HG-PEL-5 [ADW77534] correspond to *Heterodera glycines*; HS-PEL-1 [ABN14273] and HS-PEL-2 [ABN14272] correspond to *H*. *schachtii*; GR-PEL-1 [AAF80747] and GR-PEL-2 [AAM21970] correspond to *Golobedera rostochiensis*; GP-PEL-2 [ACU64826] corresponds to *G*. *pallida*; BX-PEL-1 [BAE48369] and BX-PEL-2 [BAE48370] correspond to *Bursaphelenchus xylophilus*; BM-PEL-1 [BAE48373] and BM-PEL-2 [BAE48375] correspond to *B*. *mucronatus*; AA-PEL-1 [BAI44999] and AA-PEL-2 [BAI44997] correspond to *Aphelenchus avenae*; MI-PEL-1 [AAQ09004] and MI-PEL-2 [AAQ97032] correspond to *Meloidogyne incognita*. Identical residues are highlighted in black. Black bars (I to IV) indicate the conserved regions characteristic of the PL3 pectate lyases. The asterisk (*) indicates conserved cysteine residues. The positions of the intron *hg-pel-3*, *hg-pel-4* and *hg-pel-7*are indicated by Hg, that of *hg-pel-6* by Hg6, that of *Bx-pel-1*, *Bx-pel-2*, *Bm-pel-1* and *Bm-pel-2* by B, that of *Gr-pel-1* by Gr1, that of *Mi-pel-1* by Mi1and that of *Mi-pel-2* by Mi2. Diamonds, black triangles and white triangles represent phase 0, 1 and 2 introns, respectively.

The respective lengths of the genomic sequences of *Hg-pel-3*, *Hg-pel-4*, *Hg-pel-6* and *Hg-pel-7* were 1,301 bp, 1,326 bp, 1,299 bp and 1,742 bp from the ATG to the stop codon. Comparisons of cDNA and genomic sequences showed that a single intron was present in *Hg-pel-3*, *Hg-pel-4* and *Hg-pel-6*; in contrast, *Hg-pel-7* contained 6 introns. The intron position and phase of *Hg-pel-3*, *Hg-pel-4*and *Hg-pel-6* were the same as those in *Aa-pel-2* (GenBank No. AB495307), *Bx-pel-1* (GenBank No. AB232908), *Bx-pel-2* (GenBank No. AB232909), *Bm-pel-1* (GenBank No. AB232911) and *Bm-pel-2* (GenBank No. AB232912); however, this position was not shared in *Hg-pel-7*. In addition, *Gr-pel-1* (GenBank No. AF134582) from *G*. *rostochiensis* had six introns, and all of the intron positions and intron phase were fully shared with *Hg-pel-6*. Finally, *Mi-pel-2* (GenBank No. AY515703) from *M*. *incognita* had two introns; the positions of these introns (but not the intron phase) were shared with two of the six introns of *Hg-pel-6* and *Gr-pel-1* (Fig 1).

### Sequence and phylogenetic analysis

The sequence analysis indicated that the deduced protein sequences of Hg-PEL-3, HG-PEL-4 and HG-PEL-6 shared over 91% similarity and had a best BLAST hit with HS-PEL-2 (ABN14272) of *H*. *schachtii*. These sequences shared only 25.09% and 26.20% similarity with HG-PEL-1 and HG-PEL-2 of *H*. *glycines*, respectively; these latter proteins were previously described by Boer *et al*. (2002) and Gao *et al*. (2003), respectively. The similarities among HG-PEL-7, Hg-PEL-3, HG-PEL-4 and HG-PEL-6 were only in the range of 25.46%-26.20%; however, HG-PEL-7 was more similar to HG-PEL-1 and HG-PEL-2 with 94.46% and 95.20% of similarity, respectively. The identity and similarity shared with pectate lyases from bacterial and fungal species ranged from 25% to 45% and 41% to 60%, respectively. The identity and similarity values of pectate lyases among different cyst nematodes are shown in S1 Table. All of the deduced proteins had a predicted N-terminal signal peptide for secretion. In addition, the cleavage site of Hg-PEL-3, Hg-PEL-4 and Hg-PEL-6 was predicted between Gly_20_ and Gln_21_, whereas that of Hg-PEL-7 was predicted between Ala_19_ and Gln_20_. A conserved domain search confirmed that four conserved peptides of the PL3 family at the C terminal were identified from all the novel putative pectate lyase proteins (Fig 1). This search suggests that the sequences described here can be classified as new members of the PL3 family.

**Fig 2:**
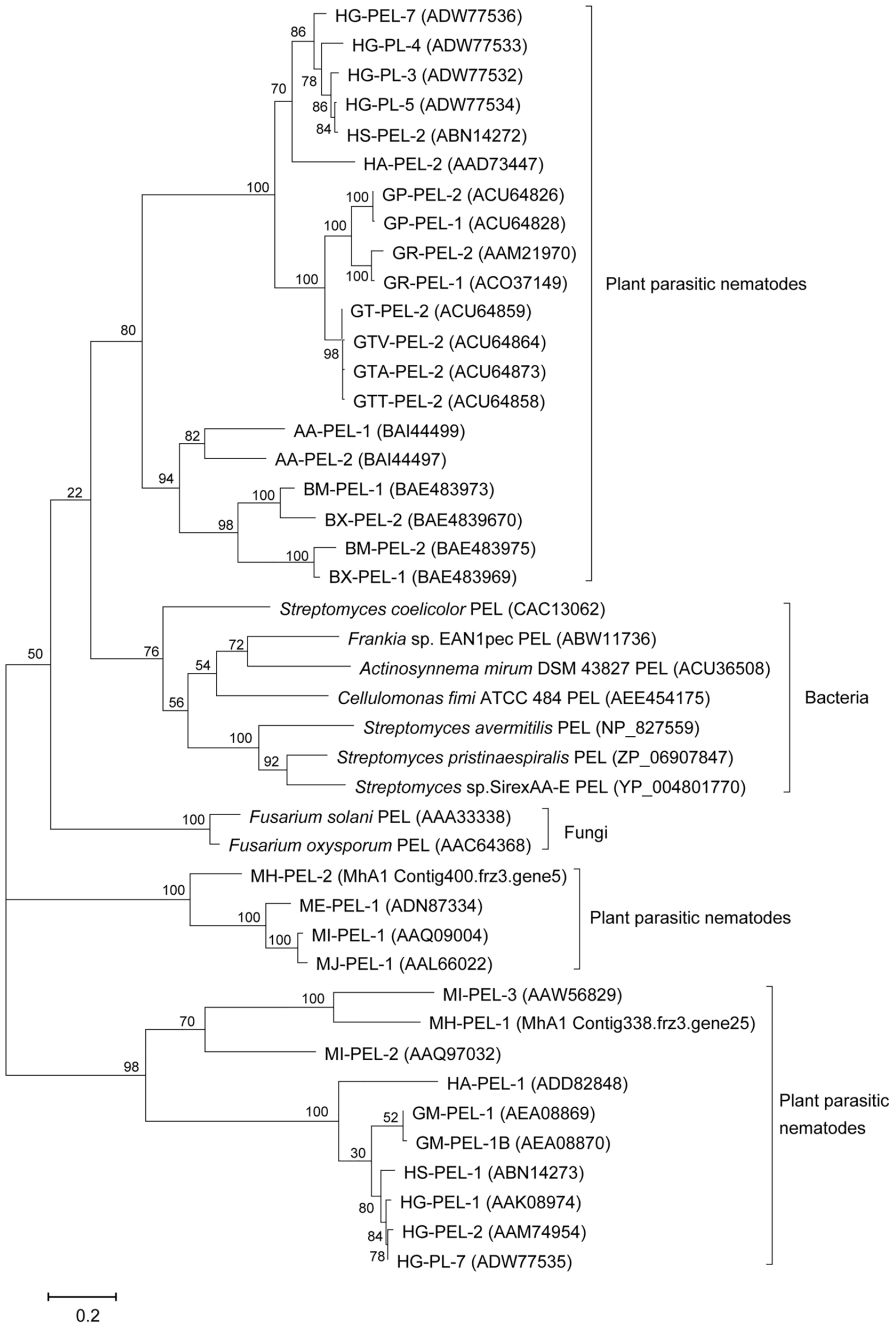
Phylogenetic tree of the polysaccharide lyase family 3 generated using maximum likelihood of PhyML. The numbers next to the branches indicate the bootstrap value. GenBank accession numbers of pectate lyase proteins correspond to *Meloidogyne incognita* (MI-PEL-1, MI-PEL-2 and MI-PEL-3), *M*. *hapha* (Mh-ENG-1 and Mh-ENG-2), *M*. *javanica* (MJ-PEL-1), *Globodera rostochiensis* (GR-PEL-1and GR-PEL-2), *G*. *pallida* (Gp-ENG-1 and Gp-ENG-2), *Heterodera glycines* (HG-PEL-1, HG-PEL-2, HG-PEL-3, HG-PEL-4, HG-PEL-5, HG-PEL-6 and Hg-ENG-7), *H*. *schachtii* (HS-PEL-1 and HS-PEL-2), *H*. *avenae* (HA-PEL-1 and HA-PEL-2), *Aphelenchus avenae* (AA-PEL-1 and AA-PEL-2), *Bursaphelenchus xylophilus* (BX-PEL-and BX-PEL-2), and *B*. *mucronatus* (BM-PEL-1 and BM-PEL-2).

A phylogenetic analysis suggested that the seven putative pectate lyases of *H*. *glycines* were classified into two different branches (Fig 2). For instance, HG-PEL-3, HG-PEL-4, HG-PEL-5 and HG-PEL-6 were clustered with HS-PEL-2 of *H*. *schachtii*, HA-PEL-2 of *H*. *avenae* and pectate lyases of *Globodera* spp., *A*. *avenae* and *Bursaphelenchus* spp. In contrast, HG-PEL-7, Hg-PEL-1 and HG-PEL-2 clustered together with HS-PEL-1 of *H*. *schachtii*, HA-PEL-1 of *H*. *avenae* and two pectate lyases of *G*. *mexicana*. However, other nematode sequences were not monophyletic; instead, they were clustered into three distinct clades.

### Southern blotting

**Fig 3:**
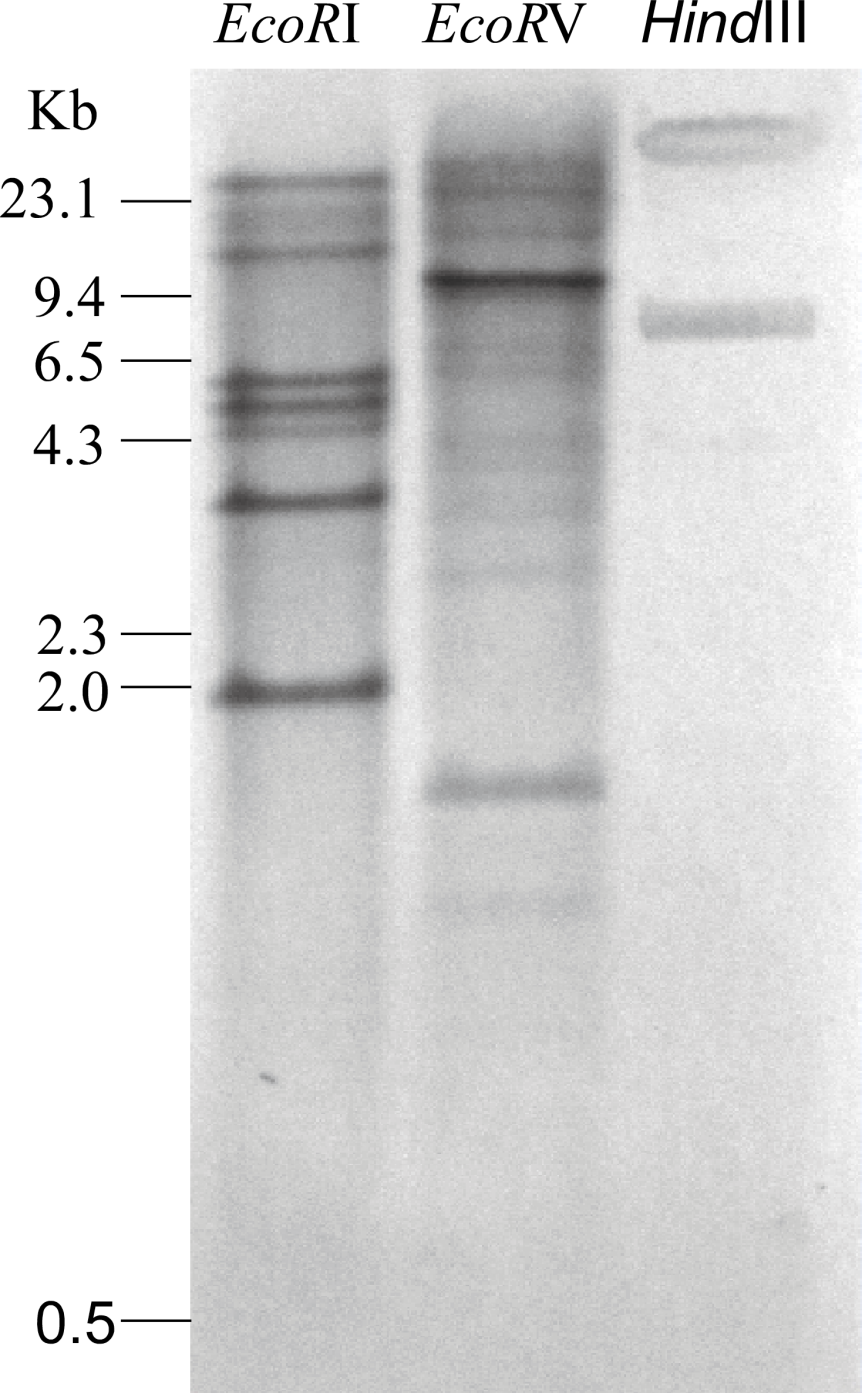
Southern blotting of *Hg-pel-6*. Southern blot of the genomic DNA from *H*. *glycines* digested with *Eco*RI, *Eco*RV and *Hind*III as well as hybridized with an *hg-pel-6* cDNA probe. The DNA size markers are indicated in kb.

A Southern blot that contained genomic DNA from *H*. *glycines* was hybridized with a DNA probe designed to specifically hybridize with *hg-pel-6* (Fig 3). Digestion with three different enzymes suggested that several related pectate lyase genes are present in the genome of *H*. *glycines*.

### *In situ* hybridization

**Fig 4:**
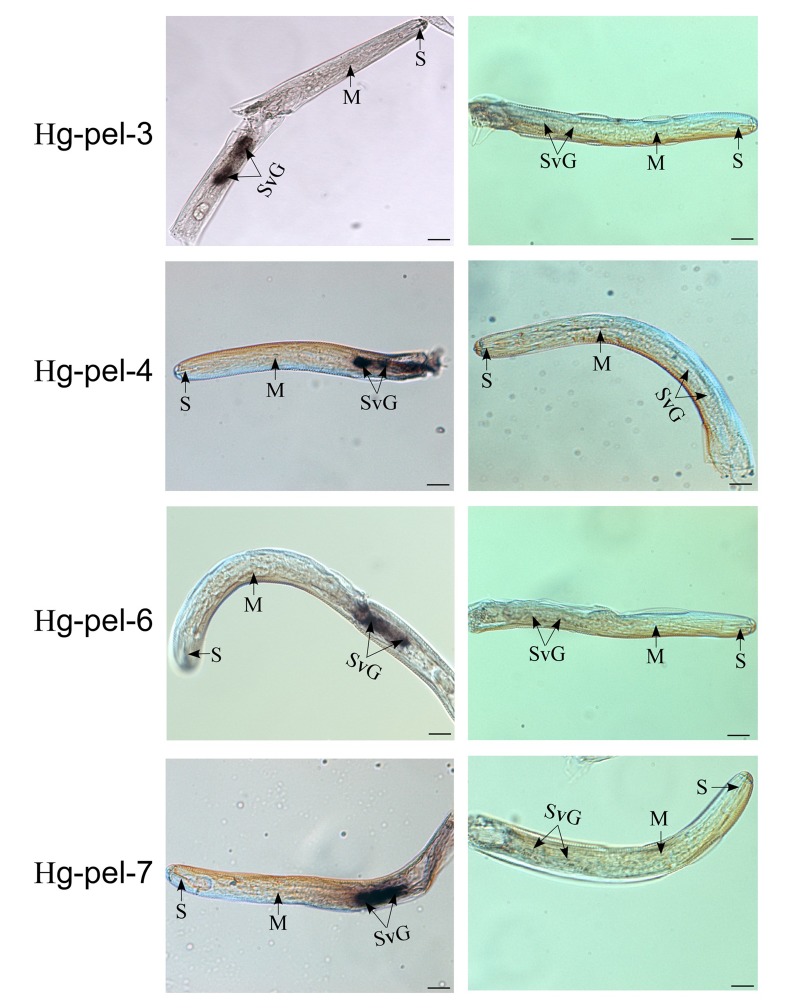
Localization by *in situ* hybridization of *hg-pel-3*, *hg-pel-4*, *hg-pel-6* and *hg-pel-7* in pre-parasitic second stages of *Heterodera glycines*. Left panel: antisense *Hg-pel-3*, *Hg-pel-4*, *Hg-pel-6* and *Hg-pel-7* probes; right panel: corresponding sense probes. S, stylet; M, median bulb; SVG, subventral esophageal cell. Scale bar: 20 μm.

The localization of *Hg-pel-3*, *Hg-pel-4*, *Hg-pel-6* and *Hg-pel-7* was analyzed in *H*. *glycines* by *in situ* mRNA hybridization (Fig 4). The result show that four transcripts of pectate lyase genes accumulated specifically within a pair of subventral gland cells by antisense cDNA probes. No signal was detected when the control sense cDNA probes were used (Fig 4).

### Developmental expression analysis

**Fig 5:**
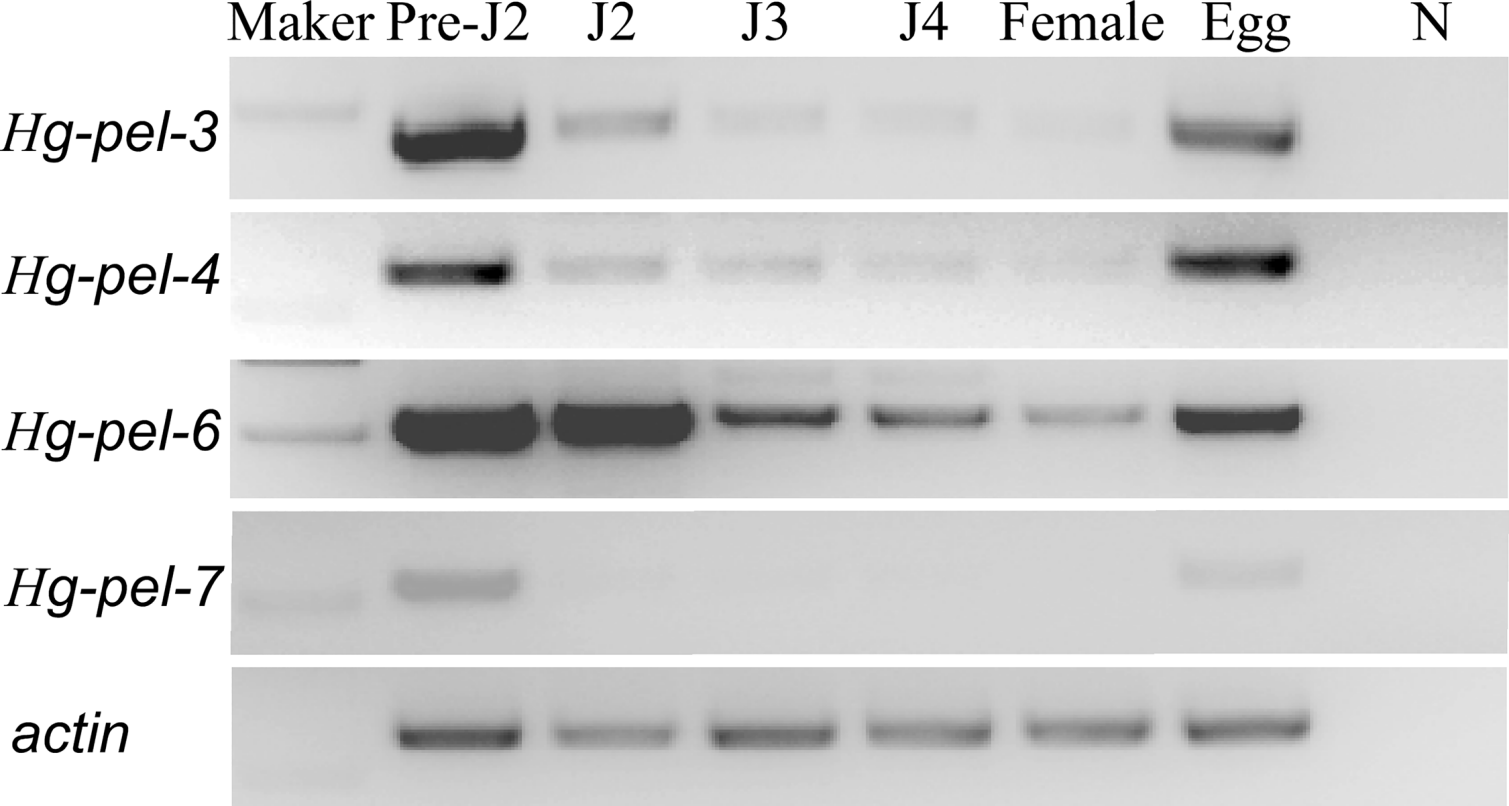
Developmental expression of *hg-pel-3*, *hg-pel-4*, *hg-pel-6* and *hg-pel-7* in different stages of *Heterodera glycines*. Pre-J2, pre-parasitic second stage juveniles; J2, parasitic second stage juveniles; J3, parasitic third stage juveniles; J4, parasitic forth stage juveniles; Female, adult females: eggs; N, negative control (genomic DNA of soybean roots). Maker, DL2000 DNA ladder. Actin, the *H*. *glycines* β-actin genem was used as positive control for each cDNA templates.

The expression levels of *Hg-pel-3*, *Hg-pel-4*, *Hg-pel-6* and *Hg-pel-7* were compared at six developmental stages, i.e., pre-parasitic J2s, parasitic J2, J3, J4, adult females and eggs, using semi-quantitative RT-PCR (Fig 5). *Hg-pel-3*, *Hg-pel-4* and *Hg-pel-7* of *H*. *glycines* were expressed at high levels in pre-parasitic J2s and eggs. Moreover, *Hg-pel-6* was expressed in eggs, pre-parasitic J2 and parasitic J2 and declined during further development of the nematode. In contrast, the expressions of the control gene (actin) were not different in different developmental stages (Fig 5).

### *In vitro* RNAi

**Fig 6:**
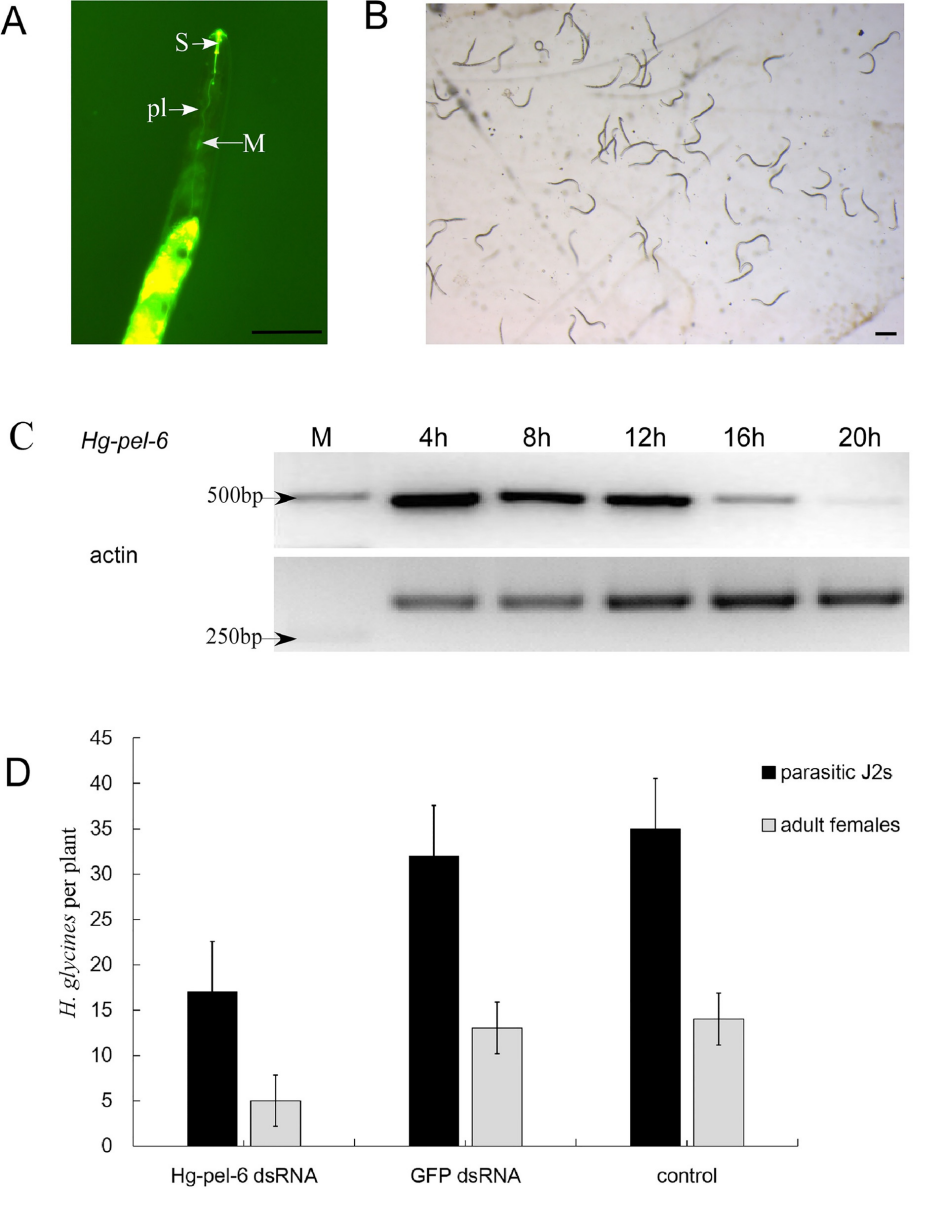
RNAi uptake of *Heterodera glycines* and infections of soybean roots with nematodes following the *Hg-pel-6* dsRNA treatment. The fluorescein isothiocyanate (FITC) uptake was observed by fluorescence microscopy (A), and the activity of the nematodes after soaking was observed under a light microscope (B). S, stylet; M, median bulb; pl, pharyngeal lumen. Scale bar: 50 μm. (C) An RT-PCR analysis for the expressions of *Hg-pel-6* and *actin* at 4 h, 8 h, 12 h, 16 h and 20 h after soaking in dsRNA targeted at *hg-pel-6*. (D) Number of *H*. *glycines* per plant at 4 dpi (black) and 30 dpi (gray) after treatment with dsRNA.

To determine the functional importance of pectate lyases during a nematode infection, *Hg-pel-6* was knocked down using *in vitro* RNAi. The uptake of materials from a solution was induced by exposure to octopamine, as confirmed by the presence of a fluorescent signal in the stylet and median bulb of *H*. *glycines* at 20 h after soaking in a buffer that contained fluorescein isothiocyanate (FITC; Fig 6A). A semi-quantitative RT-PCR analysis showed that the *Hg-pel-6* transcripts were not significantly reduced at 12 h after soaking; instead, the transcripts were increasingly suppressed at 16 h and 20 h (Fig 6B). Invasion studies showed that the treatment of J2 with *hg-pel-6* dsRNA significantly reduced (46.9%, P = 0.001) the ability of the nematodes to infect plant roots compared to nematodes that were soaked in GFP dsRNA as a control. The number of adult females established within plants decreased by 61.5% at 30 Days post inoculation (dpi, Fig 6C). There were no significant differences between GFP dsRNA-treated controls and nematodes that were not exposed to dsRNA.

### *In planta* RNAi

**Fig 7:**
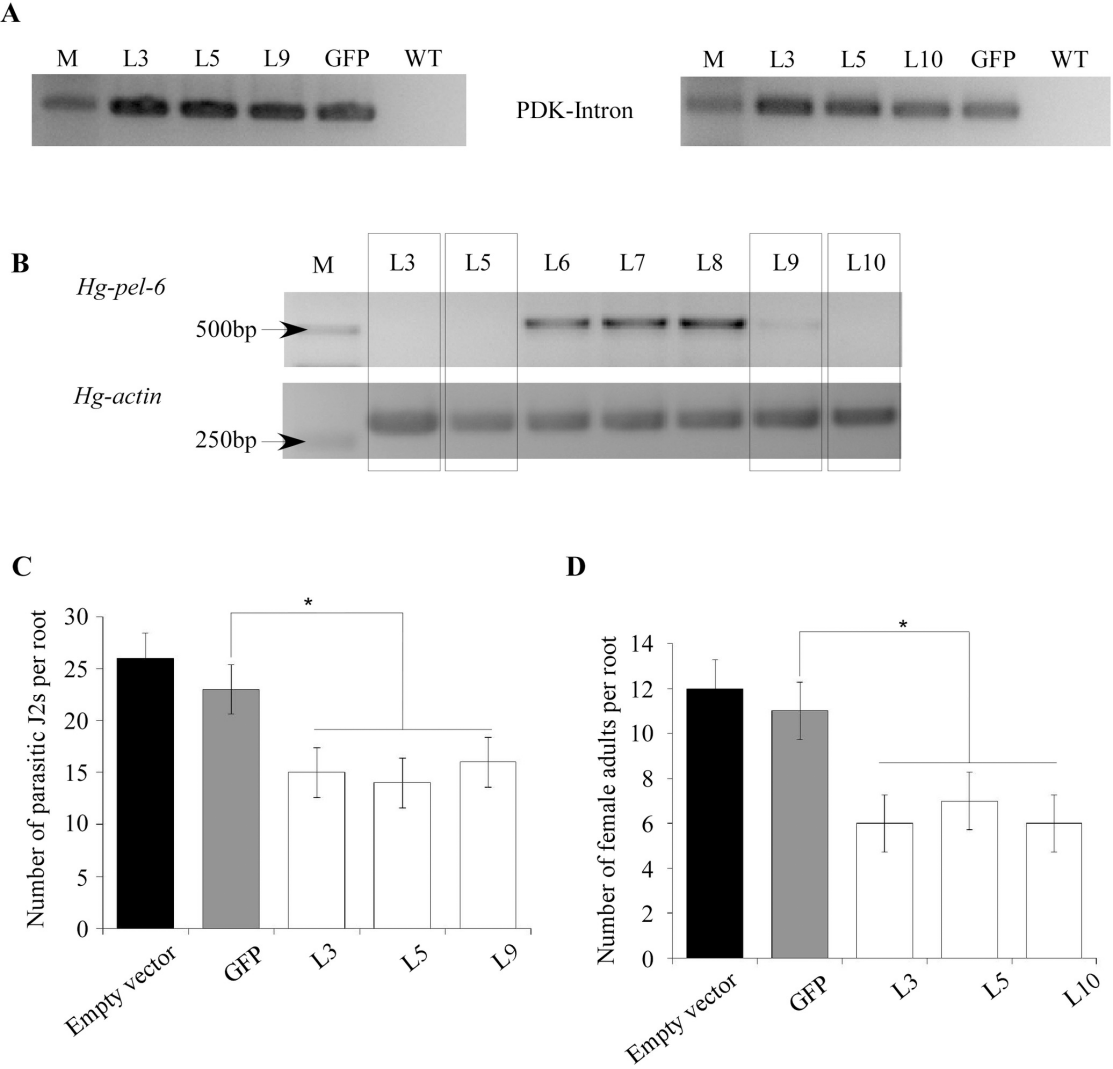
Effect of plant host-derived RNA-interference of *Hg-pel-6* on the hairy roots of soybean plants by *H*. *glycines*. (A) An RT-PCR of the single-stranded PDK intron of the hairpin dsRNA was used to confirm the expression of the hg-pel6-dsRNA and non-specific control GFP dsRNA (RNAi) in independent hairy roots of soybean lines at 10 d post-germination. (B) A semi-quantitative RT-PCR confirmed the knockdown of *Hg-pel-6* expression after an RNAi treatment of *H*. *glycines*. (C and D) Number of *H*. *glycines* per root system that expressed Hg-pel6 dsRNA at 7dpi and 40dp. The error bars indicate the standard error of the mean; n = 8. The asterisk represents a significant difference (t test, P <0.05) with respect to the control.

To confirm the results of the function analysis with *in vitro* RNAi, a representative *Hg-pel-6* cDNA sequence was used to express dsRNA *in planta* driven by the CaMV35S promoter. The hairpin constructs were confirmed by the expression of the dsRNA using RT-PCR (Fig 7A). The *Hg-pel-6* transcript level significantly declined after the treatment of *in vivo* RNAi (Fig 7B). The transgenic expression of the *Hg-pel-6* hairpin construct resulted in a 30.4–39.1% reduction in the number of nematodes compared to the GFP control at 7 dpi (Fig 7C). Furthermore, the number of adult females on the hairy roots decreased by 36.4–45.5% at 40 dpi (Fig 7D), which suggests an important role of HG-PEL-6 during parasitism.

## Discussion

In this article, we report the identification and functional analysis of four novel pectate lyase genes from *H*. *glycines*. The sequence similarity between HG-PEL-3, HG-PEL-4, HG-PEL-6, HG-PEL-7 and the published pectate lyases, i.e., HG-PEL-1 and HG-PEL-2, was relatively low, but all of the sequences contained conserved regions [21] and characteristic cysteines [8] of PL3 pectate lyases (Fig 1). Therefore, we considered them to be members of the PL3 pectate lyase family, which also includes all of the pectate lyases identified from other plant-parasitic nematodes to date [8–15].

Previous research showed that there were at least two types of pectate lyases in *H*. *schachtii* [20] and *G*. *rostochiensis* [12]; however, only one type was found in *H*. *glycines*. In this study, three of the four described sequences were significantly different from the previously cloned HG-PEL-1 and HG-PEL-2. This outcome indicates that the two types of pectate lyases were also present in genome of *H*. *glycines* and that they may play difference roles. Herron *et al*. [22] suggested that a pathogen that secretes a battery of pectinolytic enzymes would be expected to have a broad host range because each of these enzymes recognizes a different sequence of methylated and non-methylated oligo galacturonate units. In addition, our phylogenetic study revealed that the pectate lyase proteins of *H*. *glycines* were clustered into distinct clades. This result, combined with the distinct patterns of intron number and position between the two types of sequences, suggests that the pectate lyase genes of *H*. *glycines* may have evolved from at least two ancestral genes. These results are consistent with previous research, which suggests that the PL3 of plant parasitic nematodes was acquired by a few independent lateral gene transfers (LGT) events from different subfamilies [23].

The *in situ* hybridization experiments localized the mRNA of *Hg-pel-3*, *Hg-pel-4*, *Hg-pel-6* and *Hg-pel-7* in the subventral esophageal glands. These results are consistent with data from other phytopathogenic nematodes [9–15] and further confirm that the subventral gland cells of endo-parasitic nematodes function during the early parasitic process of plant invasion and migration of J2s through plant tissues [24]. The gland-cell-specific expression location together with the presence of predicted signal peptides on all the putative proteins suggest that pectate lyases possible secretion may play a key role during nematode infection. Three of the four novel pectate lyase genes are highly expressed in pre-parasitic J2s and eggs, which suggests that these genes contribute to pectin degradation and assist in the root invasion and successive migration of the nematode through the plant roots. The developmental expression pattern is similar to that previously observed in *H*. *schachtii* and *M*. *incognita* [11,13]. However, *Hg-pel-6* is also expressed during the parasitic stages, which suggests that this protein may play a key role in the latter stages of the parasitic interaction. A similar result was described for *Mi-pel-1* in *M*. *incognita*[11].

Two pectate lyases, *Hg-pel-1* and *Hg-pel-2*, were previously isolated and characterized from *H*. *glycines* [9]. Because *Hg-pel-6* and these proteins shared low similarity and because *Hg-pel-6* had a different developmental expression pattern compared to the other sequences, this gene was selected and knocked down by RNAi to investigate its functional role. RT-PCR showed that the silencing of *hg-pel-6* occurred 16 h after soaking and increased 20 h after soaking. These results confirm those of Chen *et al*. [25], who described that silencing was induced in *G*. *rostochiensis* after it was soaked for 24 h. In general, the RNAi phenotype will be enhanced with an increase in the soaking time, particularly for cyst nematodes [26]; however, the RNAi of other genes can also be carried out with 4 h of incubation in the cyst nematode [27,28].

Bakhetia *et al*. [20] reported that the silencing of *hg-pel-2* induced a change in the sexual fate that favored the male development but did not suppress the number of infections. Our results demonstrate that a reduction in the *Hg-pel-6* mRNA resulted in lower infections. Similar results have been reported in *H*. *schachtii* [13]. Furthermore, the development of adult females *H*. *glycines* was also suppressed. These results imply that *Hg-pel-6* plays an important role in the infection process. The low sequence similarity observed between *Hg-pel-6* and *Hg-pel-2* and the fact that knocking down *Hg-pel-6* and *Hg-pel-2* by RNAi induced different phenotypes indicate that the two types of pectate lyases in the soybean cyst nematode may play different roles during infection.

Our study shows that *H*. *glycines* targets pectic polysaccharides with at least two types of pectate lyase genes. Moreover, these genes may perform separate functions in different developmental stages. These novel putative pectate lyases of *H*. *glycines* are important in the infection process. These experimental results agree with the general idea that PPNs secrete a diverse range of enzymes to loosen and degrade the plant cell wall structure, thus facilitating the migration through the root tissue [8].

## Materials and Methods

### Nematode culture

*H*. *glycines* race 4 was isolated from Langfang City, Hebei Province, China. A single cyst was cultured on soybean (*Glycine max*) roots in autoclaved soil and grown in a greenhouse. Eggs were collected from *H*. *glycines* cysts, and hatched pre-parasitic J2 were collected by placing the eggs on a sieve (25 μm pore size) in 3 mM ZnSO_4_ at 25°C. To isolate the parasitic juveniles and later life stages of *H*. *glycines*, 10-day-old soybean plants were inoculated with *H*. *glycines* J2 (approximately 25,000). Infected roots were harvested at 4, 8, 14 and 30 dpi to collect parasitic J2s, J3s, J4s and adult females, respectively. These stages were isolated as previously described [29].

### DNA and RNA isolation and cDNA synthesis

Genomic DNA was isolated from approximately 5000 pre-parasitic J2s with a DNeasy Blood & Tissue Kit (Qiagen, GmbH, Germany). Total RNA was extracted with TRIzol (Invitrogen, Carlsbad, CA, USA) and treated with RQ1 RNase-Free DNase (Promega, Madison, WI, USA). mRNA was isolated from pre-parasitic J2s, parasitic J2s, J3s, J4s, adult females and eggs with a Dynabeads mRNA Direct kit (Invitrogen, Carlsbad, CA, USA). The first-stranded cDNA was synthesized with SuperScript First-Strand Synthesis System (Invitrogen, Carlsbad, CA, USA) according to the manufacturer’s instructions.

### Isolation of pectate lyases

Specific primers were designed to amplify the 3´and 5´cDNA ends of four sequences identified from *H*. *glycines* expressed sequence tags (ESTs) in the GenBank. To obtain full length cDNAs, the 5´ and 3´ cDNA ends of the pectate lyases were amplified from total RNA of pre-parasitic J2 with specific primers (Table 2) using a GeneRacer kit (Invitrogen, Carlsbad, CA, USA) according to the manufacturer’s instructions. The genomic sequences of the pectate lyases were obtained from genomic DNA with PEL3-FF, PEL3-FR, PEL4-FF, PEL4-FR, PEL6-FF, PEL6-FR, PEL7-FF and PEL7-FR. All amplicons were cloned into PMD-18 vector (TaKaRa Biotech, Dalian, China) and sequenced by the Beijing Genomics Institute.

**Table 2:** Primers used in this study.

Primer	Sequences (5’–3’)	Descriptions
PEL3-3R-1	TCAAAGATTTTGGCTTCAAACGGTT	3' RACE
PEL3-3R-2	TGGTGGGAAAAGGTTGGCGAAGACG	
PEL3-5R-1	TTGCCAATGTTTTCGGCCACAAA	5' RACE
PEL3-5R-2	TTCTTCGATGGTCCGCTGACTTG	
PEL4-3R-1	GCGGATGCAAGGAGTACAATTTTCT	3' RACE
PEL4-3R-2	AAGTGACTGGGCTGAAAACGCTATT	
PEL6-3R-1	TGATGTGAACAATGGCAAACTGAAA	3' RACE
PEL6-3R-2	GCAAAGGCACAACCATCATCAAGAA	
PEL7-3R-1	GCTACAAAAGATTGACCGCCTCTTC	3' RACE
PEL7-3R-2	AGGAGTACAATTTTCTGGTGACGGG	
PEL7-5R-1	GCGTCTTCTCCAACCTTCTCCCA	5' RACE
PEL7-5R-2	GCGTCTTCTCCAACCTTCTCCCA	
PEL3-FL-F	ATGCATTCCTTTCTTGTACTGAC	Genomic DNA amplification
PEL3-FL-R	ATTCCCTTCTCATTTTAACACTG	
PEL4-FL-F	CAATGTATTCCTTCCTTGTTCTA	Genomic DNA amplification
PEL4-FL-R	GTGGTGCTCGAAATTATGCAATT	
PEL6-FL-F	TCATCAATGTCCTTTTTACTTT	Genomic DNA amplification
PEL6-FL-R	TGAGTCTTTTGCAATCAGTTTA	
PEL7-FL-F	AATGATGCTTTTCATTCTATTGG	Genomic DNA amplification
PEL7-FL-R	TTTACTTTCATCACTCAACCCTC	
PEL3-TZ-F	TTCAAACGGTTGACCGCCTCTTC	RT-PCR and in situ hybridization
PEL3-TZ-R	TGCCACATCGCCATAATTCTCGT	
PEL4-TZ-F	AGCGGTTGACTGCCTCTTCTGCA	RT-PCR and in situ hybridization
PEL4-TZ-R	CGCCACGTCGCCATAATTCTCGT	
PEL6-TZ-F	ATTCACTGCTATGACGGATGCAC	RT-PCR and in situ hybridization
PEL6-TZ-R	GCGTTTTCAGCCCAGTCACTTTA	
PEL7-TZ-F	CTACTGGGTTCGGGTATAAGAGC	RT-PCR and in situ hybridization
PEL7-TZ-R	GTAGTTGGAATTAAGCGAGACAA	
ACT-F	CGCTGAACCCGAAGGCCAACAGA	RT-PCR
ACT-R	TTGATGTCACGGACGATCTCACG	
PEL6-T7-F	TAATACGACTCACTATAGGGATTCACTGCTATGACGGATGCAC	dsRNA synthesis
PEL6-T7-R	TAATACGACTCACTATAGGGGCGTTTTCAGCCCAGTCACTTTA	
GFP-T7-F	TAATACGACTCACTATAGGGCTTCTCGTTGGGGTCTTT	dsRNA synthesis
GFP-T7-R	TAATACGACTCACTATAGGGACAAGTTCAGCGTGTCCG	
PEL6-IR-F	CTAGTCTAGACTCGAG ATTCACTGCTATGACGGATGCAC	In vitro RNAi
PEL6-IR-R	CGCGGATCCGAATTC GCGTTTTCAGCCCAGTCACTTTA	
gfp-IR-F	CTAGTCTAGACTCGAG CTTCTCGTTGGGGTCTTT	In vitro RNAi
gfp-IR-R	CGCGGATCCGAATTC ACAAGTTCAGCGTGTCCG	
PDK-F	GACGAAGAAGATAAAAGTTGAGAG	RT-PCR
PDK-R	TTGATAAATTACAAGCAGATTGGA	

### Sequences analysis

A total of 24444 ESTs from *H*. *glycines* were downloaded from the GenBank, clustered and assembled using CAP3 [30]. BLAST analyses were performed on the non-redundant (nr) databases at the NCBI to identify the sequence similarity [31]. The ORFs of pectate lyases were analyzed using the ORF finder (http://www.ncbi.nlm.nih.gov/gorf/gorf.html). The signal peptides of proteins were predicted using SignalP 4.0 [32]; the transmembrane domain prediction was performed using TMHMM (http://www.cbs.dtu.dk/services/TMHMM/). The molecular weight (Mw) was analyzed using Compute pI/MW tool (http://web.expasy.org/compute_pi/ website). The multiple sequence alignment was performed with ClusterW. The intron regions of pectate lyases were identified using Gene Structure Display Server (GSDS) with default parameters [33]. The conserved domain searches were carried out with the mature protein sequences at the NCBI and InterProScan (www.ebi.ac.uk/InterProScan/).

### Phylogenetic Analysis

The published pectate lyase sequences were downloaded from the GenBank. The amino acid sequences without signal peptides were aligned using Muscle, alignment curation was performed withGblocks (v0.91b). The phylogeny was reconstructed from the alignment on the Phylogeny.fr platform [34] based on the maximum likelihood method (PhyML program) and reliability for internal branches was assessed using the bootstrapping method (500). A default substitution model was selected assuming an estimated proportion of invariant sites (of 0.021) and 4 gamma-distributed rate categories to account for rate heterogeneity across sites. The tree rendering was performed using TreeDyn software (v1.83) and MAGE 5.0 [35].

### Southern hybridization

The genomic DNA of *H*. *glycines* was digested with *Eco*RI, *Eco*RV and *Hin*dIII (New England Biolabs, Ipswich, MA, USA). The resulting fragments were separated by electrophoresis on a 0.8% agarose gel and transferred to a positively charged Nylon Membrane (GE Healthcare Biosciences, NJ, USA). A cDNA probe was synthesized using a PCR DIG Probe Synthesis Kit (Roche Molecular Diagnostics, Mannheim, Germany) with specific primers (PEL6-TZF and PEL6-TZR; Table 2). Pre-hybridization (in DIG Easy Hyb at 42°C for 2 h), hybridization (in DIG Easy Hyb at 42°C overnight) and immunodetection were performed following the manufacturer’s instructions using a DIG High Prime DNA Labeling and Detection Starter Kit I (Roche Molecular Diagnostics, Mannheim, Germany).

### *In situ* hybridization

An *in situ* hybridization was carried out according to de Boer *et al*. [36] and Long *et al*. [37] with minor modifications. The sense and anti-sense probes were generated by asymmetric amplification and labeled using digoxigenin-11-dUTP (Roche Molecular Diagnostics, Mannheim, Germany) with gene specific primers (PEL3-TZ-F, PEL3-TZ-R, PEL4-TZ-F, PEL4-TZ-R, PEL6-TZ-F, PEL6-TZ-R, PEL7-TZ-F and PEL7-TZ-R; Table 2). Freshly hatched J2 nematodes were washed three times with 1× phosphate-buffered saline (PBS) buffer (Sigma-Aldrich, St. Louis, MO, USA); then, they were fixed overnight in 4% paraformaldehyde at room temperature and cut into two to five pieces. The hybridization was carried out overnight at 50°C. Hybridized probes were detected with a DIG High Prime DNA Labeling and Detection Starter Kit I (Roche Molecular Diagnostics, Mannheim, Germany) according to the manufacturer’s instructions. The nematodes were examined using microscopy (Leica, DM2500, Germany).

### RT-PCR

Fragments of pectate lyases from six developmental stages of *H*. *glycines* were PCR-amplified for 30 cycles with the primers used in the above *in situ* hybridizations (Table 2). Actin (AF318603) was amplified as a positive control using the primers ACT-F and ACT-R (Table 2). The gene silencing effect was tested with an RT-PCR after soaking in dsRNA with the primers PEL6-TZ-F and PEL6-TZ-F (Table 2). The PCR products were separated on a 1.5% agarose gel and stained with Ethidium Bromide (EB).

### *In vitro* RNA interference

The RNAi method was based on that described by Urwin *et al*. [27] and Sukno *et al*. [38] with minor modifications. Briefly, the dsRNA was designed within the coding region of *Hg-pel-6* from nucleotides (298–595 bp), synthesized and purified with a MEGAscript RNAi Kit (Applied Bio-systems, Austin, TX, USA) following the manufacturer’s instructions using the primers PEL6-T7-F and PEL6-T7-R (Table 2). A dsRNA against GFP was prepared from a gfp-containing construct using primers GFP-T7-F and GFP-T7-R. The dsRNA was re-suspended in DEPC-treated water and quantified by spectrophotometry. Approximately 3000 freshly hatched J2 were soaked in 1/4×M9 buffer (43.6 mM Na_2_HPO_4_, 22 mM KH_2_PO4, 2.1 mM NaCl, 4.7 mM NH_4_Cl) that included 50 mM octopamine, 3 mM spermidine, 0.05% gelatin, and 2 mg/ml dsRNA at room temperature, in the dark on a rotator. Control samples were incubated in the same solution but without dsRNA or with dsRNA targeted against GFP. Approximately 300 J2 individuals were moved from the soaking buffer to perform RT-PCR at 4, 8, 12, 16 and 20 h after soaking. Those nematodes were washed three times in ¼ ×M9 to remove the dsRNA and stored at -80°C before being used for RNA extractions. The remaining J2s (c. 1,800) were used for infection tests as described below. All experiments were performed in triplicate.

Susceptible *Glycine max* (soybean) cv. Lee68 was sown in 8 cm pots with autoclaved soil. Approximately 300 treated nematodes were inoculated into the soybean roots as described previously [20] and cultured at 25°C. After 4 days, the soybean roots from three pots were collected and stained using acid fuchsin, as described by Bird *et al*. [39]. Then, the infected nematodes within the roots were counted under the microscope. The remaining soybean roots were harvested at 30 dpi, and the numbers of adult female were counted under the microscope. All experiments were performed in triplicate, and the results were analyzed with the SAS software (V8.3) using Duncan’s multiple range test.

### *In planta* RNA interference

Two inverted repeat sequences of *Hg-pel-6* cDNA which was used to synthesized the dsRNA in vitro RNAi was amplified and inserted into the pHANNIBAL (RNAi) vector [40] to express the hairpin dsRNA of *Hg-pel-6* in a transformed hairy roots of soybean with primers pel6-IR-F and pel6-IR-R (Table 2). A fragment of green fluorescent protein (GFP) cDNA sequence was also constructed in pHANNIBAL with primers GFP-IR-F and GFP-IR-R (Table 2) in this study as a negative control. The *Not* I fragments, containing a repeat sequence from the pHANNIBAL vector, were sub-cloned into the binary vector pART27 [41]. The constructs were transformed into *Glycine max* (soybean) cv. Williams 82 with Agrobacterium rhizogenes strain K599 as described previously [42]. The dsRNA hairpin construct in hairy roots of soybean was confirmed by RT-PCR using a specific primer set, PDK-R and PDK-R (Table 2). The positive root lines were sub-cultured and used for further inoculation.

*H*. *glycines* J2s were hatched and surface-sterilized in 0.1% chlorhexidine digluconate (Sigma-Aldrich, St. Louis, USA) and 0.5 mg ml^−1^ hexadecyltrimethylammonium bromide (CTAB, Sigma-Aldrich, St. Louis, USA) for 20 min, followed by three washes with sterile distilled water as described previously [43], then suspended in 1.5% low melting point agarose (Invitrogen, Carlsbad, CA, USA). The active nematodes were counted under a microscope after the suspension. Approximately 30 active nematodes were inoculated onto each infection point, and three inoculation points were used per root plate. Twenty plates were inoculated per line, with three independent lines for Hg-pel-6, one line for GFP and an empty vector control. Eight plates were stained at 7 dpi, and female adults on the surface of roots from same plates at 40 dpi were counted as described above. All the results were analyzed with the methods described above. This procedure was performed to obtain *H*. *glycines* feeding on RNAi over-expression plants. The transcript levels of *hg-pel-6* in the feeding stage of *H*. *glycines* were tested with an RT-PCR using the primer set PEL6-TZ-F and PEL6-TZ-R. An actin fragment of *H*. *glycines* was also used as a positive control using the primers ACT-F and ACT-R (Table 2).

## Supporting Information

S1 Table Percentages of protein similarities and identities (bold) of pectate lyases among the different cyst nematodes. (DOC)
