# Supplementary material for: Novel Pectate Lyase Genes of Heterodera glycines Play Key Roles in the Early Stage of Parasitism
Source: PLoS One. 2016 Mar 1;11(3):e0149959. doi: 10.1371/journal.pone.0149959 (PMC4773153; doi:10.1371/journal.pone.0149959)
Supplement: S1 Table — (DOC) [file pone.0149959.s001.doc]

**S1 Table Percentages of protein similarities and identities (bold) of pectate lyases among the different cyst nematodes.**

|  | HG-PEL-2 | HG-PEL-7 | HS-PEL-1 | GR-PEL-1 | HG-PEL-3 | HG-PEL-4 | HG-PEL-5 | HG-PEL-6 | HS-PEL-2 | GR-PEL-2 | GP-PEL-2 |
| --- | --- | --- | --- | --- | --- | --- | --- | --- | --- | --- | --- |
| HG-PEL-1 | 96% | 97% | 92% | 72% | 23% | 24% | 24% | 24% | 24% | 23% | 22% |
|  | **98%** | **98%** | **95%** | **87%** | **40%** | **40%** | **40%** | **39%** | **40%** | **38%** | **37%** |
| HG-PEL-2 |  | 98% | 92% | 72% | 23% | 23% | 23% | 23% | 23% | 23% | 22% |
|  |  | **99%** | **96%** | **86%** | **40%** | **40%** | **40%** | **39%** | **40%** | **38%** | **36%** |
| HG-PEL-7 |  |  | 93% | 72% | 23% | 23% | 23% | 24% | 24% | 23% | 22% |
|  |  |  | **95%** | **86%** | **39%** | **40%** | **39%** | **39%** | **39%** | **37%** | **36%** |
| HS-PEL-1 |  |  |  | 71% | 24% | 24% | 24% | 25% | 25% | 23% | 23% |
|  |  |  |  | **85%** | **40%** | **41%** | **40%** | **40%** | **40%** | **37%** | **36%** |
| GR-PEL-1 |  |  |  |  | 24% | 25% | 25% | 25% | 25% | 24% | 23% |
|  |  |  |  |  | **40%** | **40%** | **39%** | **39%** | **40%** | **39%** | **37%** |
| HG-PEL-3 |  |  |  |  |  | 90% | 96% | 90% | 96% | 64% | 67% |
|  |  |  |  |  |  | **96%** | **98%** | **94%** | **98%** | **77%** | **79%** |
| HG-PEL-4 |  |  |  |  |  |  | 90% | 88% | 90% | 63% | 66% |
|  |  |  |  |  |  |  | **96%** | **94%** | **96%** | **78%** | **80%** |
| HG-PEL-5 |  |  |  |  |  |  |  | 91% | 98% | 64% | 68% |
|  |  |  |  |  |  |  |  | **94%** | **99%** | **77%** | **79%** |
| HG-PEL-6 |  |  |  |  |  |  |  |  | 91% | 64% | 68% |
|  |  |  |  |  |  |  |  |  | **94%** | **78%** | **81%** |
| HS-PEL-2 |  |  |  |  |  |  |  |  |  | 64% | 68% |
|  |  |  |  |  |  |  |  |  |  | **77%** | **79%** |
| GR-PEL-2 |  |  |  |  |  |  |  |  |  |  | 83% |
|  |  |  |  |  |  |  |  |  |  |  | **91%** |
